# What Keeps a Man off the Panel?

**Published:** 1914-05-23

**Authors:** F. L. Nicholls

**Affiliations:** Fulbourn


*To the Editor of* The Hospital.

### What Keeps a Man off the Panel?

*To the Editor of* The Hospital.

Sir,—A few weeks back a correspondent wrote that the profession of medicine had lost ground in the eyes of the public before the advent of the panel, and, therefore, he did not think it of any use to make a difference between panel and non-panel men. This week Dr. Thomas Johnston asks the above question. To both questions it is not difficult to supply an answer, but as the latter can be quickly disposed of we will take it first. It is supplied by the attitude of practically the whole profession in the latter part of 1912, when it made it clearly understood that all that a man valued his profession for, *qua* profession, and his freedom, would be destroyed by becoming a panel man, and in the end it resolved itself purely into a matter of £ *s. d.* We know, of course, that since that time there have been a few men who, possibly from a Chinaman's point of view, have extolled in a feeble way the good they, as panel men, have been enabled to do.

Now, to the first question, the following may supply an answer. Some months ago the influential people in a county that shall be nameless thought it desirable, as it certainly was necessary, to start a county nursing association. From the first they found themselves up against a somewhat serious obstacle. Members of the profession in villages were not favourable, with a few exceptions, and on the question of the nurses acting as midwives took quite a determined stand, some few even being almost aggressive about it. The County Association who had started the scheme from the single-minded point of view of filling a necessary want to their poor neighbours, and the members of which were giving their time and money for the purpose, found themselves in a serious difficulty. If they ignored the recalcitrant members of the profession they would probably be putting their nurses in an unenviable and difficult position, especially in villages where the doctor had openly stated he would give no help in emergencies, and on the other hand they would be debarring the poor from the boon they wished to bestow upon them, of being able to be attended by a well-trained midwife in their lying-in troubles. Under these circumstances they asked that representatives of the profession should meet them and try to see what could be done to get out of the difficulty. By a misunderstanding a non-panel doctor found himself alone amongst a number of panel men who had met to appoint delegates. He heard their views, with which he strongly disagreed, and told them they had already done the profession and themselves harm by going on the panel, and they would do still more harm by opposing the nursing scheme. He endeavoured to point out to them that the profession existed for the benefit of the public, and it would be a bad day for the profession if the public came to think that it thought more of itself than of the pure practising of its art, and at the same time he pointed out to them the uselessness of trying to stem a flowing tide. He was not with them long, as he found the atmosphere uncongenial. Some time afterwards he saw it announced in the local press that the doctors and Nursing Association had come to terms, and amongst the conditions was one that the midwife should *obtain the consent of the usual medical attendant* before taking a case, if the wages of the husband exceeded 15s. a week. Now it is quite unnecessary to go into the difficulties this might entail; but think of the impossible and invidious position of a sensitive and fair-minded man, finding himself thrust into deciding whether he or a midwife shall undertake a case!

Just recently the first annual meeting of the Nursing Association has been held, and while extolling the good work and self-denial under difficulties of a devoted body of nurses, one lady, whose heart was evidently deeply concerned with the success of the undertaking, stated that this good work had been done in spite of much ignorance and prejudice. It is not necessary to be able to see through a brick wall to understand the direction in which this remark was made, and it is sad to think that, instead of hearing something said about the benefit received by co-operation with the medical profession; one cannot help feeling that the above remark was justified. The profesion may have lost ground in the opinion of the public before the advent of the panel, but is one not justified in suspecting that the man who is careless of his pledge is most likely the one who has given the public cause for this loss of regard?

The interests of panel and non-panel are so opposed that it is impossible to believe the two classes can ever be reconciled while the panel system exists; but if every individual member of the profession can be brought to see that it is the interests of the public that should and must come first, something will then have been done to regain its goodwill and high opinion.—I am, Sir, yours truly,

F. L. Nicholls, L.R.C.S.

Fulbourn, May 17, 1914.

